# Myofibroblast-Targeting Extracellular Vesicles: A Promising Platform for Cardiac Fibrosis Drug Delivery

**DOI:** 10.34133/bmr.0179

**Published:** 2025-04-11

**Authors:** Yi Wang, Hao Jiang, Qing Chen, Fei Guo, Bei Zhang, Lin Hu, Xuege Huang, Wenwen Shen, Jiapeng Gao, Wenwen Chen, Wei Xu, Zhijian Cai, Lin Wei, Min Li

**Affiliations:** ^1^ Institute of Biology and Medical Sciences, Soochow University, Suzhou 215123, China.; ^2^ Department of Nuclear Medicine, The First Affiliated Hospital of Wannan Medical College, Wuhu 241001, China.; ^3^ Department of Urology, Changhai Hospital, Naval Medical University, Shanghai 200433, China.; ^4^ Institute of Immunology and Department of Orthopedics of the Second Affiliated Hospital, Zhejiang University School of Medicine, Hangzhou 310058, China.; ^5^ State Key Laboratory of Radiation Medicine and Protection, School of Radiation Medicine and Protection and School for Radiological and Interdisciplinary Sciences (RAD-X), Soochow University, Suzhou 125123, Jiangsu, China.; ^6^ Department of Infectious Diseases, The First Affiliated Hospital of Anhui Medical University, Hefei 230032, Anhui, China.

## Abstract

Current pharmacological treatments for cardiac fibrosis are often limited by their efficacy and specificity, leading to marked side effects. Fibroblast activation protein (FAP) is specifically expressed on activated myofibroblasts (myoFbs) but not on resting cardiac fibroblasts, making it a promising target for cardiac fibrosis therapy. In this study, we engineered extracellular vesicles (EVs) conjugated with an anti-FAP single-chain variable fragment, termed αFAP-EVs, which specifically target myoFbs. Our results demonstrated that αFAP-EVs successfully targeted activated myoFbs in vitro and localized to fibrotic regions in isoproterenol-induced mouse hearts in vivo. To further enhance delivery efficiency, αFAP-EVs were combined with clodronate-loaded liposomes (αFAP-EL@CLD) to reduce liver accumulation and improve cardiac fibrotic site targeting. αFAP-EL@CLD loaded with cholesterol-methylated- and phosphorothioate-modified miR-29b (Agomir-29b) or the transforming growth factor beta 1 receptor inhibitor GW788388 significantly inhibited myoFb activation and reduced fibrosis in isoproterenol-induced mouse models. Importantly, these drug-loaded αFAP-EL@CLD vesicles exhibited high therapeutic efficacy with minimal systemic toxicity, attributed to their stability and targeted delivery capabilities. These findings suggest that αFAP-EL@CLD vesicles are promising candidates for cardiac fibrosis therapy, offering a foundation for future clinical applications.

## Introduction

Cardiovascular diseases (CVDs) are the leading cause of death globally. More than half a billion people around the world are affected by CVDs, and an estimated 12.1 million people died from CVDs in 2021 [1]. CVDs include coronary heart disease, cardiomyopathy, heart failure, rheumatic heart disease, and cerebrovascular disease. While these etiologies of CVDs are heterogeneous, a unifying stage in the progression of almost all types seems to be the development of cardiac fibrosis. Cardiac fibrosis occurs as an adaptive remodeling process after heart injuries with potential causes such as ischemia, mechanical stress, and inflammation. However, excessive pathologic fibrosis is usually considered irreversible and represents a common terminal pathway in diverse CVDs [2,3].

Cardiac fibroblasts (CFs) play a crucial role in both normal cardiac physiology and CVD as the most prevalent cell type in the heart [4]. They are protected from mechanical stress by a cross-linked extracellular matrix (ECM) framework, sustained through ECM degradation and deposition. However, within an injury microenvironment, CFs serve as key intermediary cells that respond to cardiac pathological changes by proliferating and differentiating into myofibroblasts (myoFbs). Typically, myoFbs exhibit increased expression of type I collagen, α-smooth muscle actin (α-SMA), transforming growth factor beta (TGF-β), and other factors, consequently triggering further CF activation [5–7]. This sets in motion a positive feedback loop, fostering fibrotic effects and ultimately leading to persistent pathological fibrosis. Thus, the development of novel strategies to attenuate or reverse myoFb activation holds promise for effective therapeutic interventions in various cardiac diseases.

For a considerable duration, fibroblast-targeting therapies have faced obstacles due to the absence of distinct markers exclusive to activated myoFbs, distinguishing them from quiescent CFs or other cell types [4,8]. For example, α-SMA, one of the presently acknowledged markers, exhibits high expression levels not only on activated myoFbs but also on smooth muscle cells [9]. In 2019, Aghajanian et al. identified fibroblast activation protein (FAP) as an endogenous protein marker specifically expressed on the surface of activated myoFbs. They adoptively transferred engineered chimeric antigen receptor T cells targeting FAP in vivo, leading to a significant reduction of fibrosis in injured mice [10]. This inspiring outcome underscores the potential of utilizing chimeric antigen receptor T-cell therapy in treating cardiac fibrosis and further validates FAP as a reliable marker for myoFbs, thereby providing a novel target for the development of myoFb-targeted therapies.

Extracellular vesicles (EVs) demonstrate promising potential as vehicles for the targeted delivery of therapeutic agents [11]. EVs are defined as small vesicles that are naturally released by most cell types and consist of ectosomes and exosomes. They can transport different species of lipids and proteins, as well as the RNA and DNA of parent cells to the recipient cells. The lipid bilayer membrane of EVs can protect their cargoes from plasma or RNase and promote them to be taken up by endocytosis, membrane fusion, or phagocytosis [12]. In addition, it has been reported that EVs can overcome pathophysiological barriers and more easily penetrate regions of abnormally abundant ECM, such as fibrotic areas [13,14].

**Fig. 1.**
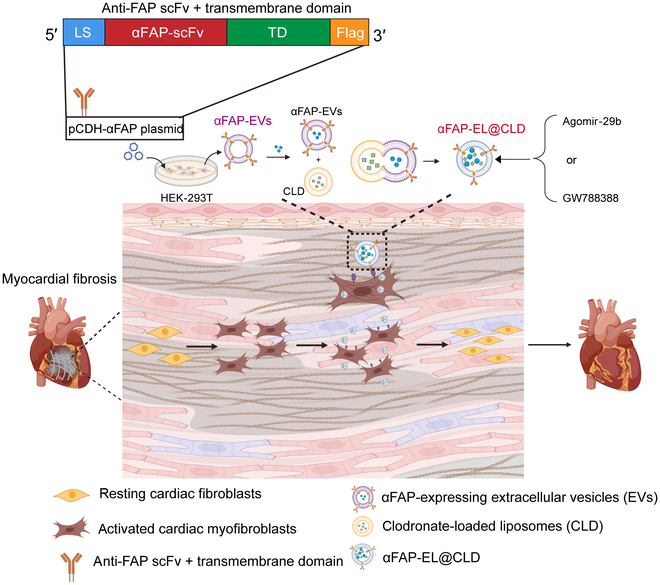
Schematic illustration of the strategy of extracellular vesicles (EVs) targeting fibroblast activation protein (FAP) and clodronate (CLD) hybrid drug delivery system for cardiac fibrosis therapy. In this study, we developed a novel hybrid system combining αFAP-expressing EVs with CLD-loaded liposomes (αFAP-EL@CLD), which could effectively target and deliver therapeutic agents to fibrotic regions in the myocardium, minimizing nonspecific liver uptake. αFAP-EL@CLD vesicles loaded with Agomir-29b or the transforming growth factor beta 1 (TGF-β1) receptor inhibitor GW788388 significantly suppressed myoFb activation and alleviated cardiac fibrosis. LS, leader sequence; TD, transmembrane domain; scFv, single-chain variable fragment.

Therefore, we developed engineered EVs specifically targeting FAP, consisting of a single-chain αFAP single-chain variable fragment (scFv) antibody (mAb 73.3) and transmembrane regions, that are capable of latching αFAP onto the EV surface (anti-FAP scFv-expressing EVs are termed as “αFAP-EVs”) (Fig. 1). The results demonstrated that αFAP-EVs could be specifically delivered into the fibrotic region in the heart, compared with con-EVs. However, more than 80% of EVs accumulate in the liver via intravenous injection. Considering that CLD-loaded liposomes have been reported to effectively reduce accumulation in the liver [15], we further constructed a hybrid system of αFAP-EV and clodronate (CLD)-loaded liposomes (αFAP-EL@CLD) through membrane fusion, and the results showed that αFAP-EL@CLD could more effectively reach fibroblastic sites in the heart. αFAP-EL@CLD vesicles loaded with cholesterol-methylated- and phosphorothioate-modified miR-29b (Agomir-29b) or the TGF-β1 receptor inhibitor GW788388 significantly inhibited myoFb activation and reduced fibrosis in isoproterenol (ISO)-induced mouse models. Notably, these drug-loaded αFAP-EL@CLD vesicles demonstrated high therapeutic efficacy with minimal systemic toxicity, which can be attributed to their stability and targeted delivery capabilities.

## Materials and Methods

### Animals and ISO-induced cardiac fibrosis model

Sprague Dawley rats (postnatal days 1 to 3) and C57BL/6J mice (6 to 8 weeks) were purchased from Shanghai Slac Animal Inc. All animal experiments were conducted in accordance with the Guide for the Use and Care of Laboratory Animals, and the study was approved by the Ethics Committee of Soochow University. To induce cardiac fibrosis, 6- to 8-week-old male C57BL/6J mice were administered ISO via subcutaneous injection at a dosage of 10 mg/kg body weight per day for 15 d. Following the 15-d ISO treatment period, the mice underwent echocardiographic examination and subsequent cardiac tissue harvesting.

### Cell culture

The HEK-293T cell line was cultured in Dulbecco’s modified Eagle’s medium (DMEM) supplemented with 2 mM glutamine, 100 units/ml penicillin, and 100 μg/ml streptomycin sulfates and 10% heat-inactivated fetal bovine serum (FBS) (Gibco) at 37 °C in the presence of 5% CO_2_.

### Plasmid construction and transfection

Using the scFv sequence of αFAP as previously reported [16], we purchased the synthesized gene sequences by incorporating a leader sequence, a transmembrane sequence, and a Flag tag (see Fig. S1) from GENEWIZ (Suzhou, China) and subcloned into the pCDH vector. HEK-293T cells were seeded into 24-well plates, and transient transfection was performed by using Lipofectamine 2000 according to the manufacturer’s instruction. The expression of αFAP was subsequently confirmed using an anti-Flag antibody.

### Isolation of EVs

EVs were harvested from HEK-293T cells using ultracentrifugation. HEK-293T cells were cultured for 48 h posttransfection in EV-free DMEM containing 10% FBS and 1% penicillin/streptomycin. The medium had been precentrifuged at 100,000×g. The culture supernatants were collected and subjected to the following sequential centrifugation steps: 300×g for 10 min, 2,000×g for 10 min, and 10,000×g for 30 min to remove cells, cellular debris, and organelles. The supernatants were then filtered through a 0.22-μm filter. EVs were pelleted by ultracentrifugation at 120,000×g for 2 h at 4 °C. The pellets were rinsed with phosphate-buffered saline (PBS), ultracentrifuged again, and resuspended in sterile PBS. Protein concentrations of the EV suspensions were determined using the bicinchoninic acid assay. Isolated EVs were stored at −80 °C. EV markers tumor susceptibility gene 101 (TSG101), CD9, CD63, and Alix were detected by Western blotting (WB) analysis.

### Preparation of EL@CLD

Clodronate disodium (CLD) was purchased from TargetMol (USA). CLD-loaded liposomes (L@CLD) were prepared using the reverse-phase evaporation method [17]. Briefly, 200 mg of L@CLD with a lipid composition of cholesterol:1,2-dioleoyl-*sn*-glycero-3-phosphocholine:1,2-distearoyl-*sn*-glycero-3-phosphoethanolamine–PEG 5000 at a molar ratio of 8:12:1 was prepared. The lipids were dissolved in chloroform, dried into a thin film on a rotary evaporator, and then resuspended in chloroform to prepare a homogeneous dispersion of the drug. A 20-ml volume of CLD solution (5 mg/ml) was gradually added to the organic phase and further emulsified under magnetic stirring for 0.5 h. The organic solvent was then removed using a rotary evaporator at 25 °C. Next, the residual lipid membrane adhering to the bottle wall was hydrated with 3 ml of PBS and extruded through 400- and 200-nm polycarbonate membranes 20 times to obtain L@CLD. The EVs and L@CLD hybrid (EL@CLD) were prepared using a membrane extrusion method. A 1-mg protein equivalent of EVs and 4 mg of lipid film were mixed to a final volume of 5 ml by vortexing and sonication (30% amplitude, 30-s pulse on/off, for 2 min) and then extruded through 400- and 200-nm polycarbonate membranes 20 times (Mini Extruder, Avanti Polar Lipids, USA).

### Western blotting

EVs were lysed in complete lysis-M buffer (Roche, Switzerland), and heart tissues were ground by liquid nitrogen and lysed in complete lysis-M buffer and then heated for 10 min at 90 °C. The concentration was determined by using the bicinchoninic acid protein assay (Thermo, Catalog [Cat.] No. 155209). An equal amount of protein was subjected to sodium dodecyl sulfate–polyacrylamide gel electrophoresis, transferred onto nitrocellulose membrane, and hybrid-blotted as described previously. Anti-Flag antibody was purchased from Abmart (Cat. No. M20008). Antibodies against collagen I (Cat. No. ab260043), CD9 (Cat. No. ab236630), CD63 (Cat. No. ab134045), TSG101 (Cat. No. ab125011), and Alix (Cat. No. ab275377) were purchased from Abcam. Anti-FAP (Cat. No. MAB9727) and anti-α-SMA (Cat. No. MAB9727) antibodies were purchased from R&D Systems. Anti-glyceraldehyde-3-phosphate (anti-GAPDH) (Cat. No. 97166), anti-total SMAD family member 3 (Smad3) (Cat. No. 9523P), anti-phospho-Smad3 (anti-pSmad3) (Cat. No. 9520P), and anti-β-tubulin (Cat. No. 86298) antibodies were purchased from Cell Signaling Technology.

### Transmission electron microscopy

A total of 5 μg of EVs or EL@CLD was diluted in PBS and placed on 200-mesh carbon-coated copper grids at room temperature for 2 min. Excess suspension was removed using filter paper. The samples were then negatively stained with uranyl acetate at room temperature for 5 min, washed twice with PBS, dried, and examined under an FEI Tecnai T10 electron microscope (FEI, Hillsboro, OR, USA) operating at 100 kV.

### Nanoparticle tracking analysis

The number and size distribution of EVs and EL@CLD were analyzed using ZetaView PMX110 (Particle Metrix, Meerbusch, German) at VivaCell Shanghai and the corresponding software ZetaView 8.04.02. Isolated EV samples were appropriately diluted using 1× PBS buffer to measure the particle size and concentration. Nanoparticle tracking analysis (NTA) measurement was recorded and analyzed at 11 positions. The ZetaView system was calibrated using 110-nm polystyrene particles.

### Flow cytometry

EVs were captured using 4-μm-diameter aldehyde/sulfate latex beads (Thermo Fisher Scientific). Briefly, 20 μg of EVs was incubated with 5 μl of beads for 30 min at room temperature in PBS (20 μl final volume). The mixture was transferred to 1 ml of PBS and shaken gently overnight. After centrifugation, the pellet was blocked with 20 μl of exosome-depleted FBS for 30 min. EV-coated beads were washed 3 times in PBS and resuspended in 50 μl of PBS. The beads were then incubated with fluorescein isothiocyanate-conjugated AffiniPure Goat Anti-Rabbit IgG (Proteintech, Cat. No. SA00003-2) in 100 μl of PBS for 30 min at room temperature. After 3 washes in PBS, the beads were analyzed by flow cytometry (BD FACSCanto II, BD Biosciences), and data were processed using the FlowJo software (TreeStar).

### Quantification of Flag in αFAP-Flag-EVs

Recombinant DYKDDDDK tag protein (contains the same sequence as FLAG Tag) was purchased from Proteintech, Wuhan, Hubei, China (Cat. No. Ag2329). αFAP-Flag-EVs (30 μg) and different amounts of recombinant Flag tag protein (1, 2, 4, 6, 8, and 10 μg) were used for sodium dodecyl sulfate–polyacrylamide gel electrophoresis, and the Flag levels in different group were tested by WB. The Flag level in 30 μg of αFAP-Flag-EVs was quantified by assaying the ratio of αFAP-Flag-EVs to 1, 2, 4, 6, 8, and 10 μg of Flag, respectively.

### Isolation of neonatal rat cardiac fibroblasts and cardiomyocytes

Primary cultures of neonatal rat cardiomyocytes (CMs) and neonatal rat cardiac fibroblasts (NRCFs) were isolated from 1- to 3-d-old Sprague Dawley rats. Ventricles were harvested, washed in 75% ethanol, and maintained in DMEM supplemented with 1 mM sodium pyruvate and 4 mM l-glutamine. Hearts were minced and digested with pancreatin (1 mg/ml in PBS without Ca^2+^ and Mg^2+^) at 37 °C for 20 min. The digestion was repeated until most cells were isolated. After each digestion, the supernatant was collected, and cells were pelleted by centrifugation. NRCFs were obtained by preplating the digested cell mixture for 2 h, allowing fibroblasts to adhere. The nonadherent CMs were then collected and diluted in DMEM:M199 (3:1), supplemented with 4.5 g/ml glucose, 10% horse serum, 5% FBS, and 100 μM bromodeoxyuridine.

### EV labeling and targeting uptake in vitro

EVs were labeled using 1,1′-dioctadecyl-3,3,3′,3′-tetramethylindodicarbocyanine perchlorate (DiD) (Beyotime, China) according to the manufacturer’s instructions. Briefly, 100 μg of EVs in 200 μl of PBS was mixed with 5 μM DiD at room temperature for 10 min. Unbound dye was then removed by ultracentrifugation at 120,000×g for 2 h, and the pellets were resuspended in 200 μl of PBS. Fluorescence analysis was conducted to assess the uptake of DiD-labeled αFAP-EVs in NRCFs and neonatal rat CMs treated with 200 μg/ml DiD-labeled αFAP-EV at various time points.

### EV tracking in vivo

Labeled EVs (100 μg, ~2.3 × 10^10^ particles) were injected into mice via the tail vein. After 24 h, the mice were sacrificed, and the heart, lungs, liver, spleen, and kidneys were collected and washed with PBS. The uptake of VivoTrack DiD-labeled EVs was imaged using an in vivo imaging system (IVIS, PerkinElmer). Background and autofluorescence were subtracted using PBS negative controls. Fluorescence images were analyzed with the Living Image software (version 4.4, Caliper Life Sciences). The radiant efficiency of the regions of interest in different organs was measured from 3 randomly selected images per organ. Exposure conditions were consistent for all measurements.

### Echocardiography

Transthoracic echocardiography was conducted on ISO-treated mice after 15 d using the Vevo 2100 high-resolution ECHO system with a 35-MHz transducer. Chest fur was removed with depilatory cream (Veet), and mice were anesthetized with isoflurane (2.5% induction, 1.0% maintenance) in oxygen. Mice were placed supine on a heated pad at 37 °C with continuous electrocardiogram monitoring to maintain heart rates between 500 and 600 bpm. The probe was positioned along the short axis of the left ventricle, and 2-dimensional images were captured to measure internal wall dimensions during systole and diastole. The left ventricular end-systolic diameter and left ventricular end-diastolic diameter were obtained from M-mode images, and the left ventricular systolic function was assessed by calculating the ejection fraction and fractional shortening. Image analysis was performed by a blinded operator using Vevo 2100 software.

### RNA extraction and quantitative PCR

Total RNA was isolated from tissues or cells with TRIzol (Accurate Biology, China). Reverse transcription was performed with Reverse Transcription Kit (Accurate Biology, China). Polymerase chain reaction (PCR) analysis was performed with Power SYBR Green (Accurate Biology, China) by using the following reaction conditions: 95 °C for 2 min followed by 40 cycles of 95 °C for 5 s and 60 °C for 34 s. For messenger RNA (mRNA) detection, GAPDH served as an internal control. The relative fold changes in the expression of mRNAs were calculated using the following equation: relative quantification = 2^−ΔΔCT^.

### Histology

Mouse heart, lung, liver, spleen, and kidney tissues were fixed in 4% paraformaldehyde and sectioned into 5-μm-thick tissue blocks. After dehydration, paraffin embedding, and serial sectioning, the sections were stained with hematoxylin and eosin (H&E), Masson’s trichrome, and Sirius Red. For H&E staining, sections were immersed in filtered Harris hematoxylin for 10 s and then in eosin for 30 s. Masson’s trichrome staining (Solarbio Science & Technology, China) and Sirius Red staining (Phygene, China) were performed according to the respective kit instructions.

### Immunohistochemistry

Murine heart tissue sections were deparaffinized, rehydrated, and subjected to antigen retrieval using 10 mM sodium citrate buffer (pH 6.0). After blocking with 5% bovine serum albumin, the slides were incubated with primary antibodies at 4 °C overnight. The sections were then incubated with horseradish peroxidase-conjugated secondary antibodies at room temperature for 30 min. Following 3 washes with PBS, the sections were stained using 3,3′-diaminobenzidine (AiFang Biological, China). Anti-FAP (Cat. No. MAB9727) and α-SMA (Cat. No. MAB9727) antibodies were purchased from R&D Systems. Images were randomly captured and analyzed using the ImageJ software (National Institutes of Health [NIH], Bethesda, MD, USA).

### Immunofluorescence

Tissues were embedded in Tissue-Tek Cryo-O.C.T. (Thermo Fisher Scientific) and sectioned at 10-μm thickness. Sections were fixed in 4% paraformaldehyde for 15 min, permeabilized with 0.3% Triton X-100 in PBS for 15 min, and blocked with 5% bovine serum albumin for 1 h at room temperature. The sections were then incubated with primary antibodies at 4 °C overnight, followed by incubation with fluorescence-labeled secondary antibodies at 4 °C for 1 h. Nuclei were stained with 0.5 μg/ml 4′,6-diamidino-2-phenylindole for 10 min at room temperature. The stained sections were observed using a Nikon A1R confocal microscope (Nikon). The areas of FAP^+^ or α-SMA^+^ cells were analyzed using the ImageJ software (NIH, Bethesda, MD, USA).

### Preparation of miRNA-loaded αFAP-EL@CLD

αFAP-EL@CLD: miR-29b was prepared using the membrane extrusion method. Briefly, 1 mg of protein-equivalent EVs was incubated with 50 nmol of Cyanine3 (Cy3)-labeled negative control miRNA (NC-miR) or miR-29b Agomir (GenePharma, China) at 37 °C for 2 h to obtain αFAP-EVs: miR-29b. Unloaded microRNA (miRNA) was removed by ultracentrifugation at 100,000×g for 2 h. Then, 1 mg of αFAP-EV: miR-29b and 4 mg of L@CLD were mixed by vortexing and sonication (30% amplitude, 30 s pulse on/off, 2 min) to a final volume of 5 ml. The mixture was extruded 20 times through polycarbonate membranes with pore sizes of 400 and 200 nm to obtain αFAP-EL@CLD: miR-29b. The encapsulation efficiency of miR-29b was determined by measuring Cy3 fluorescence in a 96-well plate using BioTek ELX800 (BioTek, USA) with excitation/emission wavelengths of 550 nm/570 nm.

### Preparation of GW788388-loaded αFAP-EL@CLD

αFAP-EL@CLD: GW788388 was prepared using the membrane extrusion method [17]. First, 1 mg of protein-equivalent EVs and 4 mg of L@CLD were mixed by vortexing and sonication (30% amplitude, 30 s pulse on/off, 2 min) to a final volume of 5 ml. Then, 10 mg of GW788388 (Shanghai Taosu BioTechnology Co., Ltd.) was added. The mixture was extruded 20 times through polycarbonate membranes with pore sizes of 400 and 200 nm, successively, to obtain αFAP-EL@CLD: GW788388.

### In vitro cumulative release of GW788388 in FBS

Con-EL@CLD: GW788388 and αFAP-EL@CLD: GW788388 were dispersed in 1 ml of FBS and incubated in a constant-temperature water bath shaker (100 rpm, 37 °C). At the time points of 0, 1, 2, 10, and 24 h, FBS containing both substances was collected. The remaining Con-EL@CLD: GW788388 and αFAP-EL@CLD: GW788388 precipitates were obtained by ultrafiltration at 4,000 rpm for 30 min. After resuspension in PBS, the samples were vacuum freeze-dried overnight. GW788388 was released from the vesicles by methanol dissolution. After centrifugation at 4,000 rpm for 10 min, the supernatant was collected. The absorption of GW788388 was detected by enzyme calibration (PerkinElmer, USA) at 260 nm. The cumulative release rate of GW788388 (%) was calculated using the following formula:

$$\begin{array}{c} \begin{array}{c} \begin{array}{c} \text{Cumulative release rate of the drug}\;\left(\%\right)=1-\\ \left[c_{n}h\;(n=0,1,2,10,24)\times V/\text{drug initial encapsulation amount}\right]\times 100\%. \end{array} \end{array} \end{array}$$ (1)

### Statistical analysis

Data are presented as mean ± standard error of the mean (SEM). Statistical comparisons were made using analysis of variance and Student *t* test. All statistical tests were conducted with GraphPad Prism version 8 (GraphPad Software, Inc., CA, USA). Significance levels are indicated as *P < 0.05, **P < 0.01, ***P < 0.001, and ****P < 0.0001.

## Results

### FAP expression is up-regulated in the myocardium of ISO mice and in CFs upon TGF-β1 stimulation

**Fig. 2.**
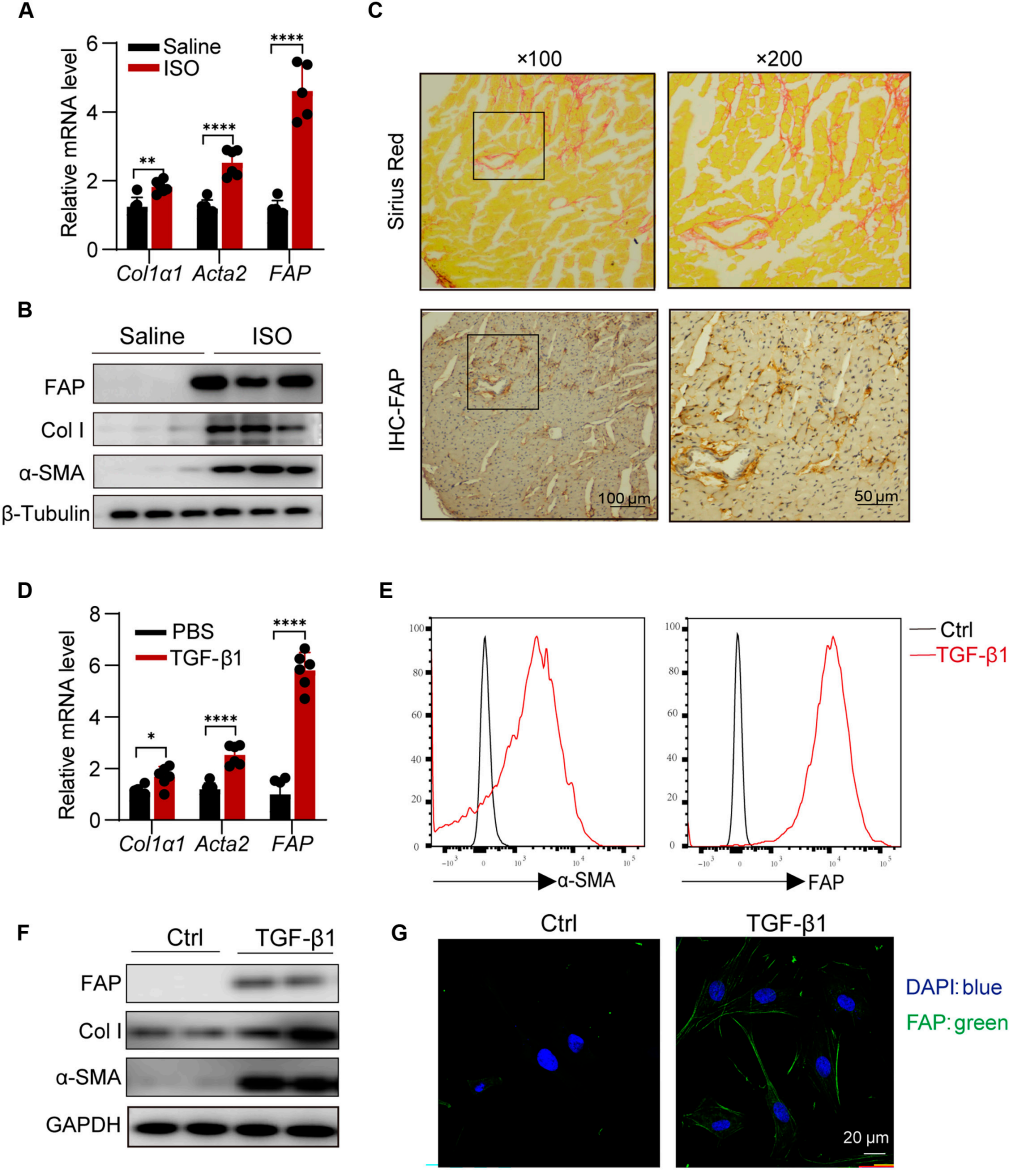
FAP expression is up-regulated in the myocardium of isoproterenol (ISO) mice and in cardiac fibroblasts upon TGF-β1 stimulation. (A) Quantitative polymerase chain reaction (qPCR) analysis showing messenger RNA (mRNA) expression levels of *Col1α1*, *Acta2*, and *FAP* in heart tissue from ISO-treated mice. *n* = 6. Data are presented as mean ± SEM. **P < 0.01; ****P < 0.0001. (B) Western blotting analysis of Col1α1, α-smooth muscle actin (α-SMA), and FAP protein expressions in the hearts of mice treated with ISO or saline. (C) Sirius Red staining and immunohistochemical staining for FAP in hearts from ISO-treated mice. (D) mRNA expression levels of *Col1α1*, *Acta2*, and *FAP* in neonatal rat cardiac fibroblasts (NRCFs) treated with TGF-β1 (10 ng/ml) for 24 h, assessed by qPCR. (E) Western blotting analysis of FAP protein expression in NRCFs after TGF-β1 treatment. (F) Flow cytometry analysis of FAP protein expression in NRCFs following TGF-β1 treatment. (G) Immunofluorescence (IF) analysis of FAP protein expression in NRCFs post-TGF-β1 treatment. IHC, immunohistochemical; Col I, type I collagen; PBS, phosphate-buffered saline; Ctrl, control; GAPDH, glyceraldehyde-3-phosphate; DAPI, 4′,6-diamidino-2-phenylindole.

Firstly, we established a mouse model of ISO-induced cardiac fibrosis. Quantitative PCR (qPCR) and WB analysis further confirmed elevated levels of FAP expression in ISO-induced heart tissues at both mRNA and protein levels (Fig. 2A and B). Immunohistochemical analysis of serial sections showed that FAP expression aligned with sites of positive Sirius Red staining, indicating that FAP is specifically expressed in the fibrotic areas of the heart (Fig. 2C).

We then isolated and cultured primary CFs and stimulated them with TGF-β1 to induce differentiation into activated myoFbs. It was found that TGF-β1 stimulation significantly up-regulated the expression of collagen I, α-SMA, and FAP (Fig. 2D and E). Flow cytometry analysis also showed a significantly increased expression of FAP at the cell surface of myoFbs following stimulation, similar to the increase in α-SMA (Fig. 2F). These findings were further corroborated by confocal microscopy results (Fig. 2G). Based on these results, we aimed to develop a drug delivery strategy targeting FAP for cardiac fibrosis treatment.

### Characterization of FAP scFv-EVs

**Fig. 3.**
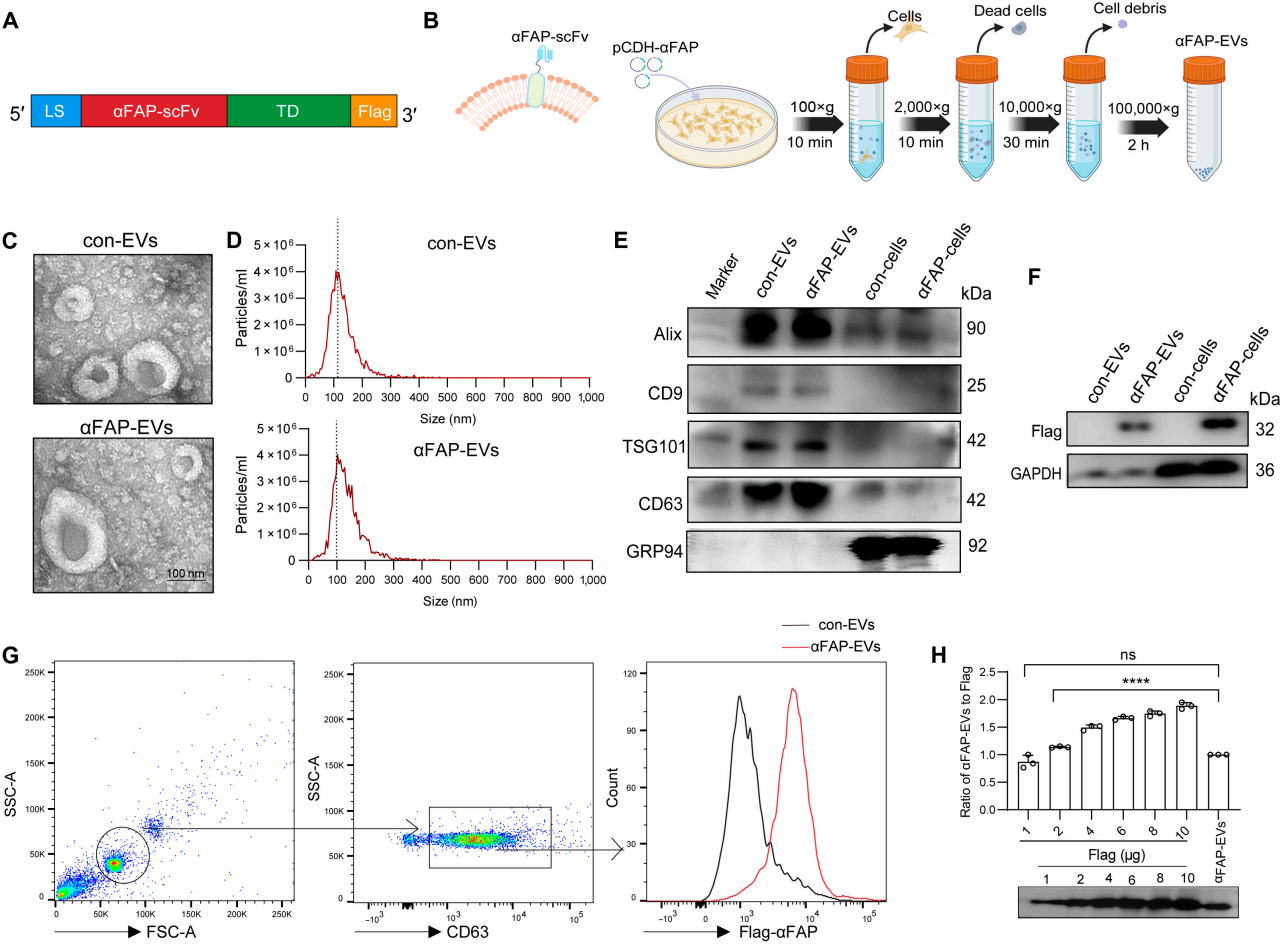
Characterization of FAP scFv-EVs. (A) Schematic diagram of the scFv FAP fusion protein. (B) Schematic representation of the preparation process of αFAP-EVs. (C) Transmission electron microscopy (TEM) images of αFAP-EVs. Scale bar = 100 nm. (D) Nanoparticle tracking analysis (NTA) of αFAP-EVs, illustrating size distribution and concentration. (E) Western blotting analysis of αFAP-EVs and αFAP-expressing cells for EV marker proteins (Alix, CD63, tumor susceptibility gene 101 [TSG101], and CD9) and non-EV marker protein GRP94. (F) Western blotting analysis for Flag-tagged proteins in αFAP-EVs and αFAP-expressing cells. (G) Flow cytometry analysis detecting the expression of CD63 and Flag in αFAP-EVs. (H) αFAP-Flag-EVs (30 μg) and different amounts of recombinant Flag tag protein (1, 2, 4, 6, 8, and 10 μg) were used for sodium dodecyl sulfate–polyacrylamide gel electrophoresis, and the Flag levels in different group were tested by Western blotting. The Flag level in 30 μg of αFAP-Flag-EVs was quantified by assaying the ratio of αFAP-Flag-EVs to 1, 2, 4, 6, 8, and 10 μg of Flag, respectively. con-EVs, control EVs; SSC-A, side scatter-area; FSC-A, forward scatter-area.

To facilitate the targeting of FAP-positive myoFbs by EVs, we designed the plasmid pCDH-αFAP. This plasmid encodes a chimeric protein that includes the following components: (a) a leader sequence, (b) an anti-FAP scFv antibody (clone 73.3) for FAP targeting, connected by a flexible (GGGS) ×3 linker, (c) a transmembrane domain derived from receptor tyrosine-protein kinase erbB-2, and (d) a Flag tag, as depicted in Fig. 3A. Figure S1 illustrates the amino acid composition of αFAP, indicating an estimated molecular weight of 32 kDa. HEK-293T cells were transfected with pCDH-αFAP, and the EVs in the culture supernatants were subsequently purified (Fig. 3B). Transmission electron microscopy (TEM) and NanoSight analysis unveiled that the EVs displayed a characteristic spherical morphology, with diameters spanning from 50 to 150 nm (Fig. 3C and D). Equal amounts of protein derived from EVs or whole-cell lysates underwent analysis via WB. The results demonstrated that both control EVs (con-EVs) and αFAP-expressing EVs (αFAP-EVs) showed similar expression levels of EV-associated proteins, including Alix, CD9, TSG101, and CD63, but did not express the endoplasmic reticulum marker GRP94 (Fig. 3E). Detection with an anti-Flag antibody confirmed the presence of the anticipated 32-kDa band in both αFAP-expressing cells and their releasing EVs, while this band was absent in the extracts of cells transfected with a mock plasmid and their corresponding EVs (Fig. 3F). Furthermore, EVs were labeled with anti-CD63 and anti-Flag antibodies and subjected to flow cytometry analysis. As depicted in Fig. 3G, the findings revealed comparable levels of EV-associated CD63 across all types of EVs, while only αFAP-EVs exhibited expression of Flag-αFAP, but not con-EVs. Furthermore, to quantify the amount of αFAP-Flag protein expressed on the EVs, we performed WB analysis using purified Flag protein as a standard. As shown in Fig. 3H, 30 μg of αFAP-EVs approximately contained 1 μg of Flag-αFAP.

### Targeted delivery of αFAP-EVs into activated myoFbs in vitro

**Fig. 4.**
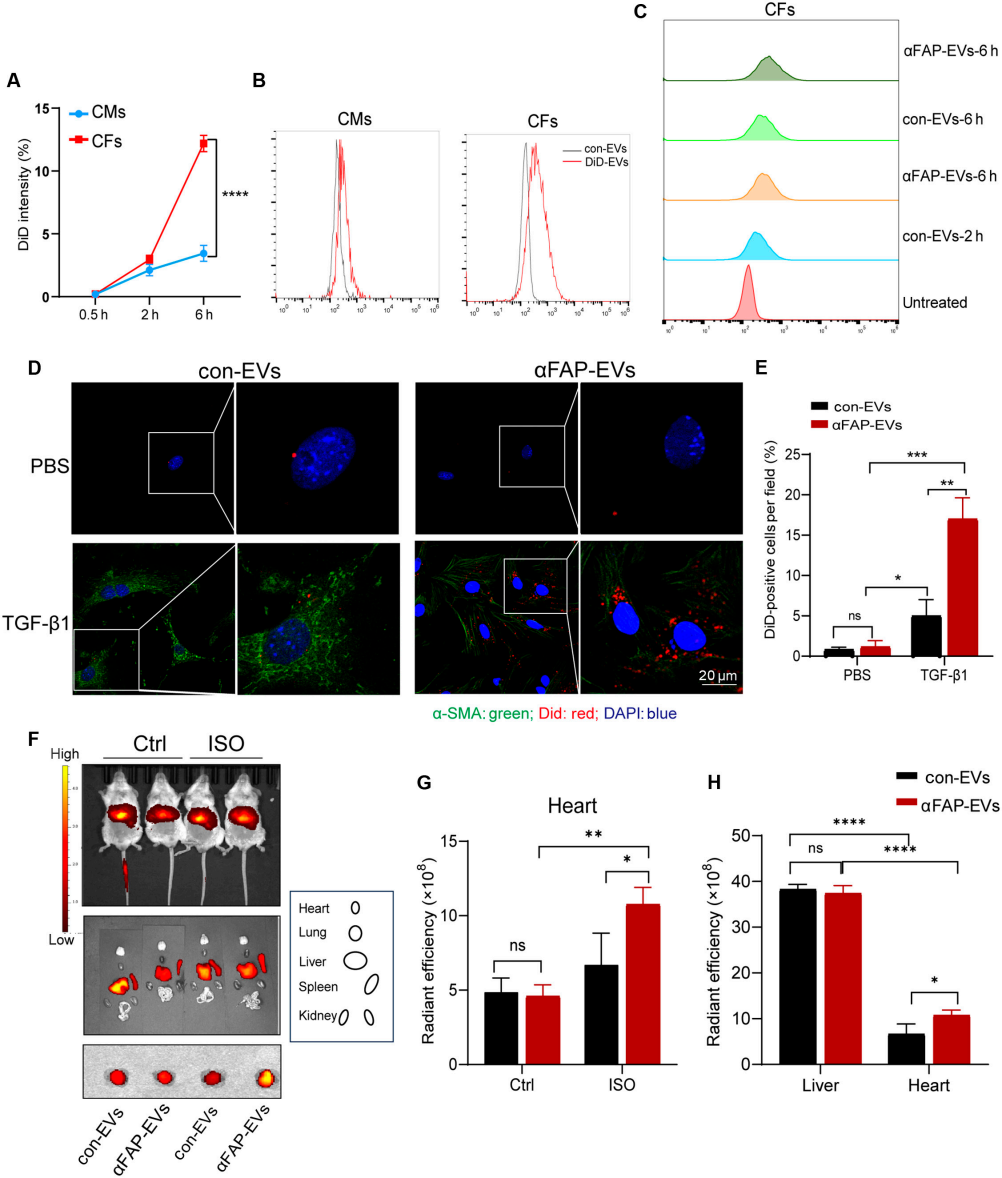
Targeted delivery of αFAP-EVs into activated myofibroblasts in vitro. (A to C) Confocal fluorescence and flow cytometry analysis of 1,1′-dioctadecyl-3,3,3′,3′-tetramethylindodicarbocyanine perchlorate (DiD) uptake in neonatal rat cardiomyocytes (NRCMs) and neonatal rat cardiac fibroblasts (NRCFs) treated with 100 μg/ml DiD-labeled αFAP-EVs at the indicated time points (*n* = 3). Quantification of mean DiD fluorescence intensity using the ImageJ software (A). Flow cytometry analysis was performed to assess the extent of DiD internalization by cells (B and C). (D) NRCFs were treated with TGF-β1 for 24 h, followed by treatment with 50 μg/ml of red fluorescent DiD-labeled con-EVs or αFAP-EVs. Activation of NRCFs was assessed by α-SMA staining. Scale bar = 20 μm. (E) Quantification of EV uptake by NRCFs using mean intracellular fluorescence intensity, analyzed with ImageJ software. (F to H) In vivo distribution of 100 μg of DiD-labeled con-EVs or αFAP-EVs injected via the tail vein into ISO-treated mice, detected by noninvasive bioluminescence imaging 24 h postinjection. Fluorescence images were captured (F), and the fluorescence intensity in the ISO model group and control group was measured (G and H). CMs, cardiomyocytes; CFs, cardiac fibroblasts.

To assess the potential targeting capacity of αFAP-EVs toward myoFbs, we labeled the EVs with DiD dye and evaluated the efficiency of cellular uptake. As shown in Fig. S2 and Fig. 4A, primary CMs and CFs were isolated, revealing a significant difference in EV uptake. CFs exhibited a notably higher signal intensity after 6 h of incubation with DiD-labeled EVs. To quantify DiD-EV uptake more accurately, flow cytometry analysis was performed. As shown in Fig. 4B and C, CFs internalized significantly more DiD-labeled αFAP-EVs than CMs and also showed higher uptake of αFAP-EVs compared to control EVs (con-EVs), supporting preferential targeting of CFs.

To corroborate the targeting specificity of αFAP-EVs toward myoFbs, CFs were treated with TGF-β1 to induce their transformation into activated myoFbs. Immunofluorescence (IF) analysis demonstrated significant up-regulation of α-SMA upon TGF-β1 stimulation, indicative of myoFb activation. Interestingly, compared to con-EVs, αFAP-EVs exhibited enhanced uptake by myoFbs, as evidenced by a conspicuous increase in red fluorescence signal intensity, while no discernible difference was observed between con-EVs and αFAP-EV (Fig. 4D and E). These findings collectively suggest that αFAP scFv tethered to the surface of EVs augments their recognition and internalization by myoFbs.

### Targeted delivery of αFAP-EVs into the hearts of ISO-treated mice in vivo

To assess the in vivo targeting efficacy of αFAP-EVs toward cardiac fibrosis sites, we initially established an ISO-induced cardiac fibrosis model. Subsequently, 100 μg of DiD-labeled con-EVs and αFAP-EVs, respectively, were intravenously administered via the tail vein. Biodistribution analysis of EVs was conducted 24 h postinjection using an IVIS imaging system. The results indicated a notably heightened fluorescence intensity within the hearts of ISO-induced cardiac fibrosis mice compared to that of the control counterparts, regardless of whether con-EVs or αFAP-EVs were administered, aligning with our in vitro findings suggesting enhanced phagocytosis by myoFbs relative to resting CFs (Fig. 4D and E). Particularly, the red fluorescence intensity of αFAP-EVs within the hearts of ISO-induced cardiac fibrosis mice exhibited a statistically significant augmentation compared to that of the con-EVs-injected group. However, no discernible difference in EV distribution was observed between the 2 groups within the hearts of control mice (Fig. 4F and G). These findings collectively underscore the effective targeting capacity of αFAP-EVs toward cardiac fibrosis sites. Nevertheless, fluorescence imaging of major organs revealed substantial EV accumulation in the livers and spleens of mice (Fig. 4H), potentially affecting the efficacy of subsequent drug delivery endeavors.

### Preparation and characterization of αFAP-EL@CLD

**Fig. 5.**
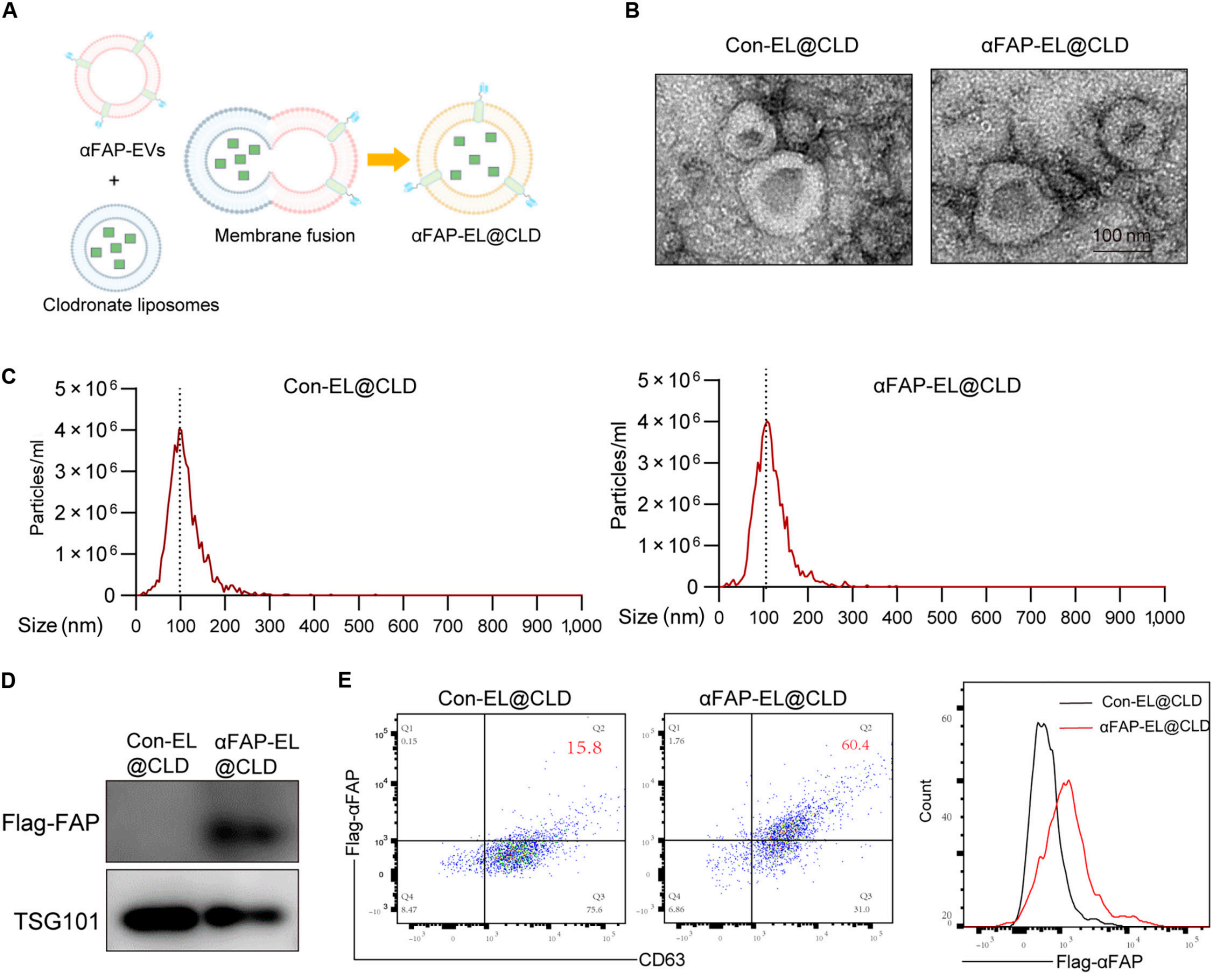
Preparation and characterization of αFAP-EL@CLD. (A) Schematic illustration of the preparation of αFAP-EL@CLD through the hybridization of αFAP-EVs with L@CLD using membrane extrusion. (B) TEM images showing the shape and size of αFAP-EL@CLD. (C) NTA of αFAP-EL@CLD, indicating particle size distribution and concentration. (D) Western blotting analysis detecting the expression of exosomal marker proteins and Flag in αFAP-EL@CLD. (E) Flow cytometry analysis of αFAP-EL@CLD, showing the expression of CD63 and Flag.

Previous reports have highlighted the capacity of CLD-loaded liposomes to induce apoptosis in Kupffer cells and significantly reduce exosome accumulation in the liver following intravenous administration [15]. Consequently, we employed membrane fusion techniques to combine prepared αFAP-EVs with CLD-loaded liposomes (L@CLD), resulting in the formation of CLD-loaded EV-liposome (EL@CLD) hybrids (Fig. 5A). TEM and NTA) revealed that the synthesized nanoparticles maintained a circular vesicle structure, with diameters ranging from approximately 100 to 200 nm (Fig. 5B and C). Subsequent WB analysis was performed to evaluate the expression of EV marker proteins TSG101 and αFAP. The results demonstrated that both the Con-EL@CLD and αFAP-EL@CLD hybrid systems retained TSG101 marker proteins post-membrane fusion. Notably, successful expression of the Flag protein was exclusively observed in αFAP-EL@CLD, with band sizes consistent with those of the anti-FAP scFv fusion protein (Fig. 5D). Additionally, flow cytometry analysis indicated positive expression of CD63 protein in the majority of Con-EL@CLD and αFAP-EL@CLD samples, confirming the preservation of EV characteristics within the EL@CLD hybrid system. Furthermore, a greater proportion of αFAP-EL@CLD exhibited Flag protein expression compared to Con-EL@CLD, implying successful expression of the membrane-type anti-FAP scFv in αFAP-EL@CLD (Fig. 5E).

### αFAP-EL@CLD enhances accumulation in the fibrotic regions of ISO-treated mice

**Fig. 6.**
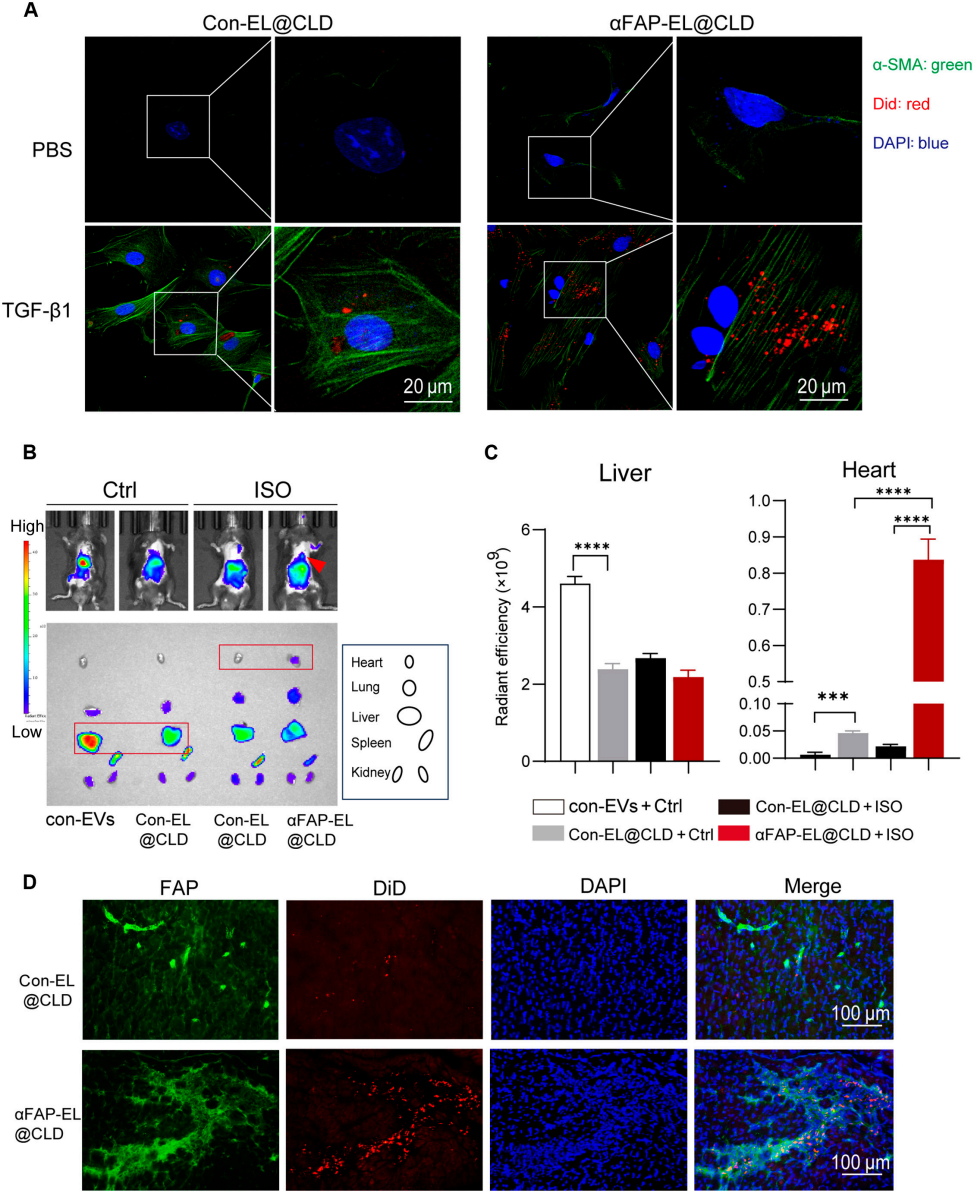
αFAP-EL@CLD enhances accumulation in the fibrotic regions of ISO-treated mice. (A) Confocal analysis of NRCFs treated with TGF-β1 for 24 h, followed by the addition of 50 μg/ml red fluorescent DiD-labeled Con-EL@CLD or αFAP-EL@CLD. Activation of NRCFs was evaluated by α-SMA staining. Scale bar = 20 μm. (B and C) In vivo fluorescence distribution of 100 μg of DiD-labeled Con-EL@CLD or αFAP-EL@CLD injected into the tail vein of ISO-treated mice, detected by noninvasive bioluminescence imaging 24 h postinjection (B). Quantification of fluorescence intensity in the hearts and livers of each group (C). (D) Confocal immunofluorescence analysis of heart tissues from ISO-treated mice, showing FAP antibody staining (green), DiD (red), and DAPI (blue) for nuclei.

Next, to investigate whether the hybrid system could exhibit enhance uptake by activated myoFbs, we labeled Con-EL@CLD and αFAP-EL@CLD with red fluorescent DiD and then coincubated them with CFs or activated myoFbs. The results revealed a significant increase in the red fluorescence intensity of activated myoFbs compared to resting CFs. Importantly, the efficiency of the phagocytosis of αFAP-EL@CLD by activated myoFbs was markedly higher than that of Con-EL@CLD (Fig. 6A), indicating that αFAP expression facilitates the targeting of αFAP-EL@CLD to myoFbs.

To further assess the targeting ability of αFAP-EL@CLD toward ISO-induced cardiac fibrosis sites, we intravenously injected DiD-labeled Con-EL@CLD and αFAP-EL@CLD into mice via the tail vein, and 24 h later, we examined the distribution of red fluorescence in vivo using the IVIS imaging system. The results indicated a significant reduction in the accumulation of Con-EL@CLD or αFAP-EL@CLD in the liver compared to that of EVs. Notably, the level of distribution of αFAP-EL@CLD in the hearts of fibrotic mice was notably higher than that of Con-EL@CLD (Fig. 6B and C). Subsequently, we harvested hearts from ISO-induced fibrotic mice and performed IF assay. The findings revealed that the FAP-positive areas exhibited red fluorescent DiD-labeled Con-EL@CLD and αFAP-EL@CLD. Importantly, the aggregation of αFAP-EL@CLD in the FAP-positive areas was significantly increased compared to that of Con-EL@CLD (Fig. 6D). These results suggest that optimizing αFAP-EVs into the αFAP-EL@CLD hybrid system significantly reduced liver accumulation, thereby enhancing targeted delivery to cardiac FAP-positive sites in fibrotic disease.

### αFAP-EL@CLD loaded with Agomir-29b inhibits myoFb activation in vitro

**Fig. 7.**
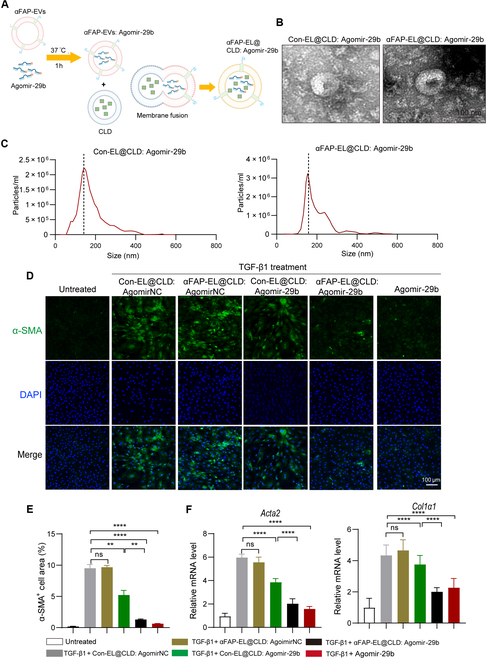
αFAP-EL@CLD loaded with Agomir-29b inhibits myoFb activation in vitro. (A) Schematic illustration of the procedure used to produce αFAP-EL@CLD loaded with miR-29b (αFAP-EL@CLD: miR-29b). (B) TEM images showing the shape and size of Con-EL@CLD: miR-29b and αFAP-EL@CLD: miR-29b. (C) NTA indicating the size and size distribution of Con-EL@CLD: miR-29b and αFAP-EL@CLD: miR-29b. (D and E) NRCFs were treated with 10 ng/ml TGF-β1 for 48 h, followed by treatment with 50 μg/ml αFAP-EL@CLD: miR-29b at 24 h. The activation level of NRCFs was evaluated by detecting the fluorescence intensity of α-SMA at 48 h. (E) Histogram of α-SMA fluorescence-positive area statistics. (F) Quantitative PCR analysis of the mRNA levels of *Acta2* and *Col1α1* in each treatment group.

Currently, microRNA-29 (miR-29) has been reported to be a novel diagnostic and therapeutic target of cardiac fibrosis [18]. MiR-29b alleviated cardiac fibrosis and improved heart function by directly targeting TGF-β2 and matrix metallopeptidase 2 (MMP2), inhibiting the activation of the extracellular signal-regulated kinase (ERK)/mitogen-activated protein (MAP) kinase triggered by angiotensin II, or up-regulating SH2B3 [19,20]. However, there are few miR-29b-based drugs to clinically treat cardiac fibrosis due to the lack of an efficient and stable cellular delivery system. So, to examine whether αFAP-EL@CLD system could make them drug delivery vehicles for cardiac fibrosis, we synthesized Agomir-29b, a cholesterol-methylated- and phosphorothioate-modified miR-29b, which has good stability and enhanced cellular uptake in vivo [21]. Then, AgomirNC or Agomir-29b was loaded into Con-EL@CLD or αFAP-EL@CLD, respectively, as illustrated in Fig. 7A. The morphological dimensions were subsequently determined by TEM and NTA and revealed that Con-EL@CLD: Agomir-29b and αFAP-EL@CLD: Agomir-29b retained a vesicular morphology, exhibiting a consistent diameter of approximately 150 nm (Fig. 7B and C). Furthermore, employing Cy3 to fluorescently label miR-29b facilitated the assessment of miRNA loading efficiency in αFAP-EL@CLD: Agomir-29b, which was determined to be approximately 10% (Fig. S3).

Initially, we sought to ascertain whether αFAP-EL@CLD: Agomir-29b could effectively impede the activation of CFs in vitro. Confocal microscopy analysis revealed that both Con-EL@CLD and αFAP-EL@CLD loaded with AgomirNC were ineffective in curtailing the TGF-β1-induced up-regulation of α-SMA expression, whereas those loaded with Agomir-29b markedly attenuated α-SMA expression. Notably, αFAP-EL@CLD: Agomir-29b exhibited a more pronounced inhibitory effect compared to Con-EL@CLD: Agomir-29b, approaching the efficacy of a 10-fold higher concentration of free Agomir-29b alone (Fig. 7D and E). Subsequently, we assessed the mRNA levels of activation-associated markers *Acta2* and *Col1α1* (a downstream effector of fibrotic signaling) via qPCR. The results showed that relative to the Con-EL@CLD: Agomir-29b treatment, the αFAP-EL@CLD: Agomir-29b treatment exhibited a significant decrease in *Acta2* and *Col1α1* mRNA expression in activated CF cells (Fig. 7F). These findings collectively indicate that αFAP-EL@CLD: Agomir-29b exerts a notable inhibitory effect on the activation of CFs.

### αFAP-EL@CLD loaded with Agomir-29b protects against cardiac fibrosis

**Fig. 8.**
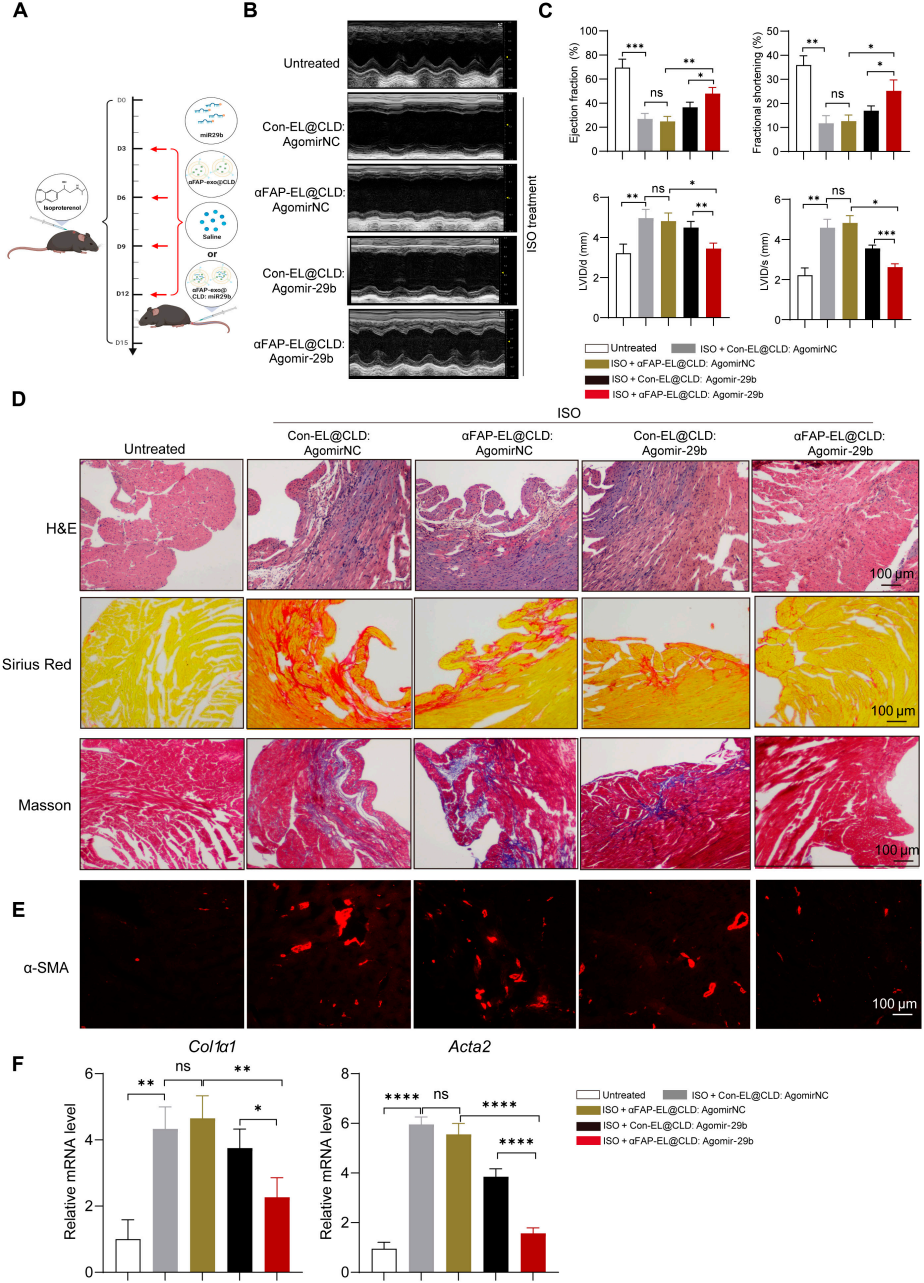
αFAP-EL@CLD loaded with Agomir-29b protects against cardiac fibrosis. (A) Schematic diagram illustrating the treatment process of ISO model mice using αFAP-EL@CLD loaded with miR-29b (αFAP-EL@CLD: miR-29b). (B and C) After 15 d of treatment, echocardiography and M-mode measurements were performed to assess cardiac systolic and diastolic functions in mice, including left ventricular shortening fraction (FS), ejection fraction (EF), left ventricular end-systolic diameter (LVID/s), and left ventricular end-diastolic diameter (LVID/d). (D) Histological analysis of mouse hearts after 15 d of treatment, using hematoxylin and eosin (H&E) staining, Masson’s trichrome staining, and Sirius Red staining to evaluate the degree of cardiac fibrosis. (E) α-SMA immunofluorescence staining was observed via confocal analysis. (F) Quantitative PCR analysis of the mRNA levels of *Acta2* and *Col1α1* in the ISO-treated mouse hearts.

To further evaluate the prospects of αFAP-EL@CLD: Agomir-29b in clinical application, we also investigated its therapeutic effects on ISO-induced cardiac fibrosis mice. αFAP-EL@CLD: Agomir-29b was administered at 3, 6, 9, and 12 d after ISO induction (Fig. 8A). The findings revealed that the left ventricular fractional shortening and ejection fraction, which reflected LV systolic–diastolic function, were markedly decreased in Con-EL@CLD: or αFAP-EL@CLD: Agomir-29b-treated mice, compared to the Con-EL@CLD: or αFAP-EL@CLD: AgomirNC-treated group, suggesting miR-29b as a therapeutic target for cardiac fibrosis. Importantly, Agomir-29b loaded into αFAP-EL@CLD showed more improved cardiac function than that loaded into Con-EL@CLD, indicating a significant decline in left ventricular systolic–diastolic function (Fig. 8B and C). Furthermore, histological analyses revealed pronounced myocardial injury in mice treated with Con-EL@CLD: or αFAP-EL@CLD: AgomirNC, characterized by conspicuous inflammatory cell infiltration, extensive collagen deposition within the cardiac interstitium, and marked fibrosis, while Con-EL@CLD: or αFAP-EL@CLD: Agomir-29b inhibited cardiac inflammation and fibrosis. Notably, in mice treated with αFAP-EL@CLD: Agomir-29b, there was a remarkable reduction in cardiac inflammation and fibrosis compared to that in mice treated with Con-EL@CLD: Agomir-29b (Fig. 8D). Correspondingly, the results of IF and qPCR also demonstrated that the expressions of α-SMA and collagen I were obviously decreased after administration with αFAP-EL@CLD: Agomir-29b, relative to those for Con-EL@CLD: Agomir-29b (Fig. 8F). All of the above findings suggest that miR-29b possesses the capacity to delay cardiac fibrosis progression. Moreover, αFAP modification significantly enhanced the efficacy of αFAP-EL@CLD: Agomir-29b treatment compared to that of Con-EL@CLD: Agomir-29b.

### αFAP-EL@CLD loaded with GW788388 protects against cardiac fibrosis

**Fig. 9.**
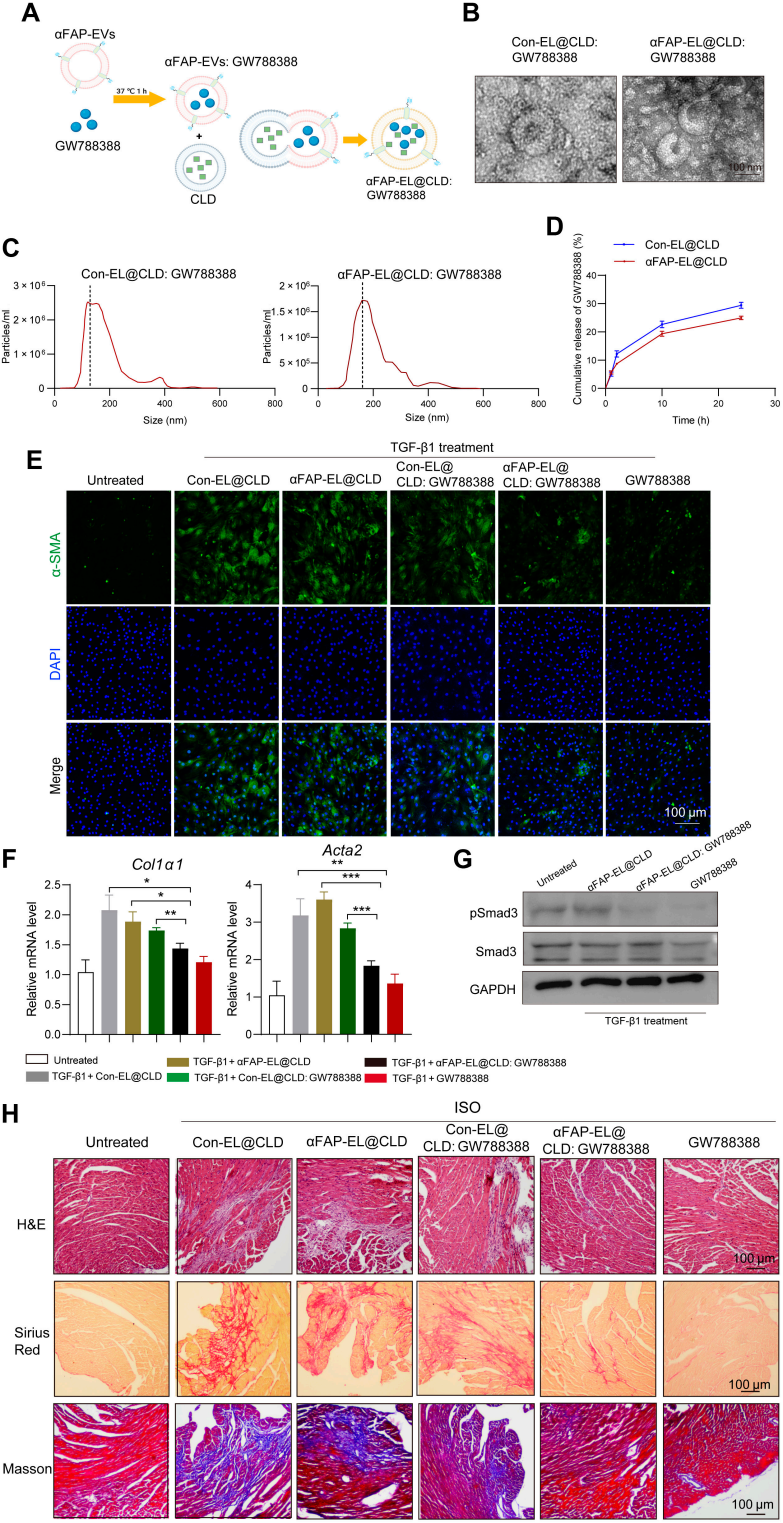
αFAP-EL@CLD loaded with GW788388 protects against cardiac fibrosis. (A) Schematic illustration of the procedure used to produce αFAP-EL@CLD loaded with GW788388 (αFAP-EL@CLD: GW788388). (B) TEM images showing the shape and size of Con-EL@CLD: GW788388 and αFAP-EL@CLD: GW788388. (C) NTA indicating the size and size distribution of Con-EL@CLD: GW788388 and αFAP-EL@CLD: GW788388. (D) The cumulative release curves of GW788388 in Con-EL@CLD and αFAP-EL@CLD in FBS (*n* = 3). (E to G) NRCFs were treated with 10 ng/ml TGF-β1 for 48 h, followed by treatment with 50 μg/ml αFAP-EL@CLD: GW788388 at 24 h. (E) The activation level of NRCFs was evaluated by detecting the fluorescence intensity of α-SMA at 48 h. (F) Quantitative PCR analysis of the mRNA levels of *Acta2* and *Col1α1* in each treatment group. (G) The phosphorylation of SMAD family member 3 (Smad3) was detected by Western blotting. (H) Histological analysis of mouse hearts after 15 d of treatment, using H&E staining, Masson’s trichrome staining, and Sirius Red staining to evaluate the degree of cardiac fibrosis.

GW788388 is a selective inhibitor of TGF-β type I receptor kinase activity, which has been demonstrated to attenuate fibrosis in various preclinical models. This compound’s efficacy in inhibiting TGF-β signaling pathways makes it a promising therapeutic candidate for conditions characterized by pathological fibrosis, such as liver fibrosis and diabetic nephropathy [22,23]. To further validate the effectiveness of αFAP-EL@CLD as a delivery vehicle for therapeutic drugs targeting cardiac fibrosis, we assessed the therapeutic effect of αFAP-EL@CLD loaded with GW788388 (Fig. 9A). Initially, the physical characteristics of αFAP-EL@CLD: GW788388 were evaluated. TEM and NTA revealed that both αFAP-EL@CLD and αFAP-EL@CLD: GW788388 displayed typical saucerlike bilayer morphologies with a mean diameter of 150 nm (Fig. 9B and C). Next, the loading efficiency of GW788388 by αFAP-EL@CLD was assessed. Based on the standard curve for GW788388, it was determined that 100 μg of αFAP-EL@CLD encapsulated approximately 13.3 μg of GW788388 (Fig. S4). To assess the in vitro cumulative release of GW788388 in serum, the αFAP-EL@CLD: GW788388 complex was incubated with serum at 37 °C for various time points (1, 2, 4, 8, 12, and 24 h). FBS containing the complex was collected at each time point. The cumulative release curves of GW788388 from Con-EL@CLD: GW788388 and αFAP-EL@CLD: GW788388 were analyzed. As shown in Fig. 9D, αFAP-EL@CLD encapsulation facilitated sustained drug release over time. The cumulative release rate from αFAP-EL@CLD was slightly lower than that of Con-EL@CLD. This result might be attributed to the αFAP coating, further confirming the successful construction of αFAP-EL@CLD.

The inhibitory effect of αFAP-EL@CLD: GW788388 on the activation of CFs was then examined in vitro. Confocal microscopy analysis showed that neither Con-EL@CLD nor αFAP-EL@CLD up-regulated TGF-β1-induced α-SMA expression, while those loaded with GW788388 significantly attenuated α-SMA expression (Fig. 9E). Notably, 50 μg of αFAP-EL@CLD: GW788388 exhibited a more pronounced inhibitory effect compared to Con-EL@CLD: GW788388, approaching the efficacy of free GW788388. Correspondingly, qPCR data indicated that TGF-β significantly up-regulated the mRNA levels of pro-fibrotic genes, including *Acta2* and *Col1α1* (Fig. 9F). Additionally, we also examined the effect of αFAP-EL@CLD: GW788388 on the TGFβ signaling pathway. As shown in Fig. 9G, αFAP-EL@CLD: GW788388 significantly inhibited Smad3 phosphorylation, consistent with previous reports.

A cardiac fibrosis model was established by administering ISO, and the mice were intravenously injected with 100 μg of αFAP-EL@CLD: GW788388. As expected, Con-EL@CLD and αFAP-EL@CLD had no therapeutic effect on cardiac fibrosis progression. In contrast, αFAP-EL@CLD: GW788388 demonstrated a marked inhibitory effect, significantly stronger than that of Con-EL@CLD: GW788388. A similar inhibitory effect was observed with free GW788388, although it required a higher drug concentration than that loaded in αFAP-EL@CLD (Fig. 9H). These results indicate that αFAP-EL@CLD: GW788388 effectively alleviates the progression of cardiac fibrosis. A similar inhibitory effect was also obtained by free GW788388, which was far more the drug concentration of αFAP-EL@CLD loaded. Therefore, these data indicate that the αFAP-EL@CLD: GW788388 could efficiently alleviate the progression of cardiac fibrosis.

### Toxicity evaluation of drug-loaded αFAP-EL@CLD

**Fig. 10.**
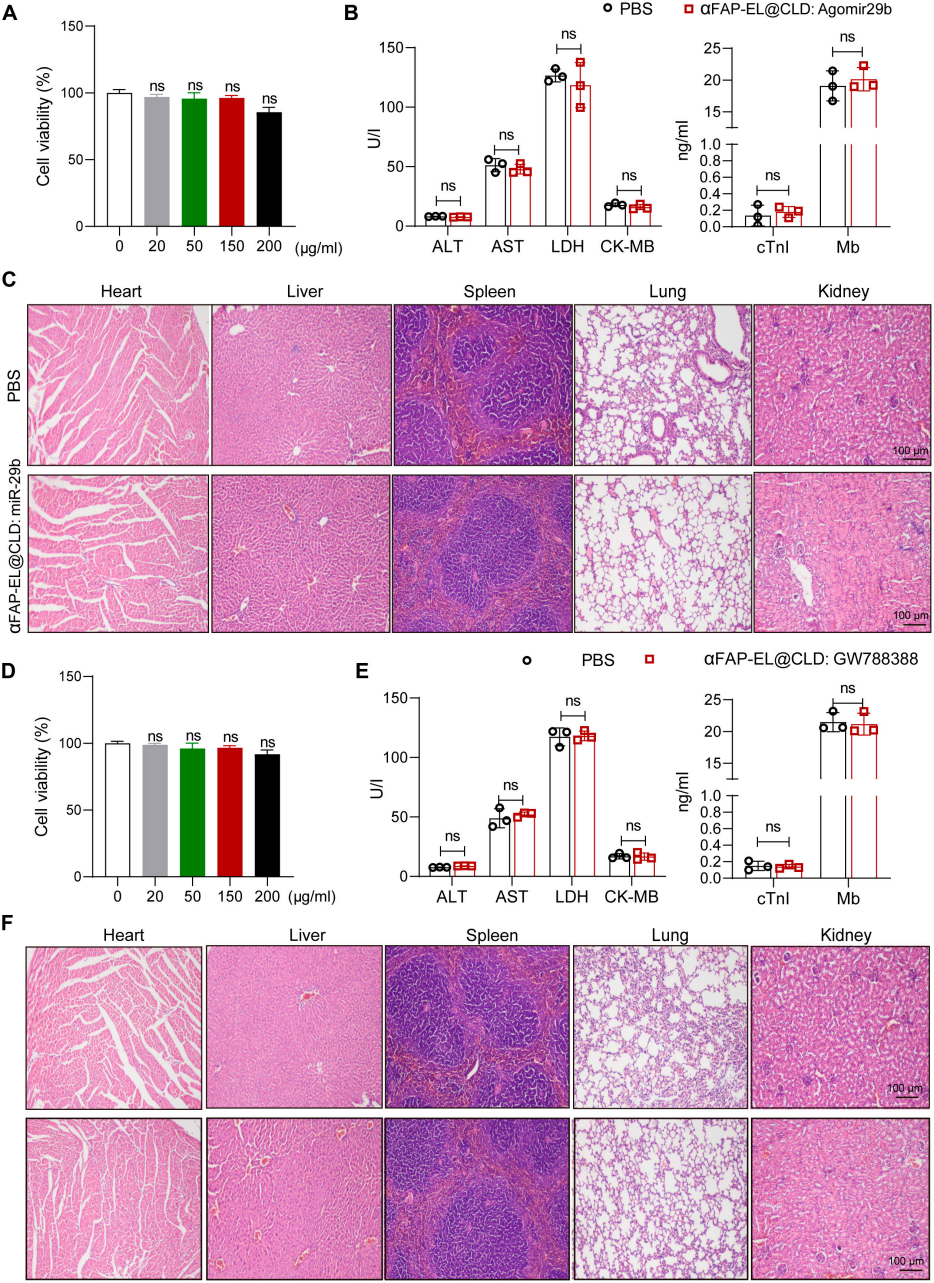
Toxicity evaluation of drug-loaded αFAP-EL@CLD. (A) After treatment with αFAP-EL@CLD: miR-29b at different concentrations for the indicated time, NRCF cell viability was measured by a Cell Counting Kit-8 (CCK-8) assay. (B) Mice were intravenously injected with 100 μg of αFAP-EL@CLD: miR-29b (≈2.3 × 10^10^ particles) on days 1, 2, and 3 and were sacrificed on day 7. The levels of alanine aminotransferase (ALT), aspartate aminotransferase (AST), kinase isoenzyme-MB (CK-MB), lactate dehydrogenase (LDH), cardiac troponin I (cTnI), and myoglobin (Mb) in sera were measured. (C) Histopathological damage in the heart, liver, spleen, lungs, and kidneys was detected by H&E staining. (D) After treatment with αFAP-EL@CLD: GW788388 at different concentrations for the indicated time, NRCF cell viability was measured by a CCK-8 assay. (E) Mice were intravenously injected with 100 μg of αFAP-EL@CLD: GW788388 (≈2.3 × 10^10^ particles) on days 1, 2, and 3 and were sacrificed on day 7. The levels of ALT, AST, CK-MB, LDH, cTnI and Mb in sera were measured. (F) Histopathological damage in the heart, liver, spleen, lungs, and kidneys was detected by H&E staining.

Ensuring biological safety is critical for the clinical application of any drug. Initially, we assessed the toxicity of αFAP-EL@CLD: Agomir-29b, and as shown in Fig. 10A, αFAP-EL@CLD: Agomir-29b showed no cytotoxicity in primary CFs. Then, we assessed the toxicity of αFAP-EL@CLD: Agomir-29b in mice, administering a dosage of 5 mg/kg every other day for 1 week. Serum samples were collected to measure aspartate aminotransferase, alanine aminotransferase, lactate dehydrogenase, kinase isoenzyme-MB, and cardiac troponin I. As depicted in Fig. 10B, there was no significant hepatic, cardiac, or renal toxicity observed in the αFAP-EL@CLD: Agomir-29b-treated group compared to the PBS-treated group. Furthermore, we examined H&E-stained sections of major organs, including the heart, lung, liver, spleen, and kidney, from both the αFAP-EL@CLD: Agomir-29b- and PBS-treated groups. No structural damage was evident in the αFAP-EL@CLD: Agomir-29b group (Fig. 10C).

We also conducted similar experiments to evaluate the toxicity of αFAP-EL@CLD: GW788388, finding no cytotoxicity in cultured CFs and no increase in alanine aminotransferase, aspartate aminotransferase, kinase isoenzyme-MB, and creatinine levels (Fig. 10D and E). Additionally, no significant histopathological damage was observed in the primary organs of either group of mice (Fig. 10F). In assessing the long-term effects of αFAP-EL@CLD: GW788388 in vivo, no significant hepatic, cardiac, or renal toxicity was observed up to 40 d post-administration (Fig. S5). These findings indicate that drug-loaded αFAP-EL@CLD does not cause systemic adverse effects when used for treating fibrotic diseases.

## Discussion

Current pharmacological interventions for cardiac fibrosis, including angiotensin-converting enzyme inhibitors, angiotensin II receptor blockers, and mineralocorticoid receptor antagonists, often have limited efficacy and significant side effects due to their lack of target specificity [24–26]. EVs have emerged as promising tools in drug delivery, enhancing the bioavailability and targeting of therapeutic agents [11].

The surface of EVs can be engineered to display targeting peptides or antibodies, expressed as fusions with membrane-associated domains, to achieve precise tissue targeting. For example, cardiac homing peptide-conjugated cardiac stem cell-derived exosomes have shown potential in promoting cardiac regeneration post-myocardial infarction by improving targeted delivery and therapeutic efficacy [27]. Furthermore, the targeted inhibition of myoFb activation has been emerging as more crucial for the amelioration of cardiac fibrosis. The discovery and specificity of FAP in distinguishing activated myoFbs from resting CFs in cardiac fibrosis is a significant advancement [10]. It becomes markedly up-regulated in response to cardiac injury and is associated with disease states such as hypertrophic and dilated cardiomyopathy, making it an ideal target for selective fibrosis therapies.

In this study, we engineered HEK-293T cells to express the fusion protein of scFv-FAP (αFAP) to produce myoFb-targeting enhanced EVs. To assess the topology of the scFv FAP fusion protein and its motifs (scFv and Flag tag) on the surface of the EVs, we relied on an established design strategy that includes a transmembrane sequence from the human ERBB2 gene. This sequence effectively anchors the scFv sequence on the extracellular side of the membrane, ensuring its proper surface localization for targeting [28]. Due to the absence of suitable antibodies for detecting FAP-scFv, a Flag tag sequence was introduced for easy identification and quantification. Flow cytometry analysis confirmed the expression of the αFAP-Flag fusion protein on the cell surface (Figs. 3E and 5E), and this was further corroborated by experiments showing that the EV surface expressed Flag proteins, with αFAP-EVs displaying Flag on the surface compared to control EVs. Furthermore, to quantify the amount of αFAP-Flag protein on the EVs, we performed WB analysis and estimated that approximately 1 μg of Flag protein is present in 30 μg of αFAP-EVs (Fig. 3H).

Our data confirmed that functional αFAP-EVs could target fibrotic regions within the myocardium more effectively than con-EVs. Despite this targeting ability, a significant portion of EVs accumulated in the liver due to nonspecific uptake by Kupffer cells. Sun et al. [15] investigated that exosomes hybridized with CLD-loaded liposomes could inhibit phagocytosis nonspecifically and thus significantly reduce accumulation in the liver [15,29]. So we prepared a hybrid system of αFAP-EVs and CLD-loaded liposomes (αFAP-EL@CLD), and our data revealed that much greater amounts of αFAP-EL@CLD could accumulate in FAP-positive fibrotic heart tissues. To further strengthen the reliability of our conclusions and address this limitation, we conducted in vitro experiments to provide direct evidence of αFAP-EL@CLD vesicle uptake. Primary CFs were isolated and differentiated into myoFbs using TGF-β1 treatment. Flow cytometry analysis was then performed to quantify the uptake of αFAP-EL@CLD versus control EVs. The results clearly show that activated myoFbs uptake significantly more αFAP-EL@CLD vesicles compared to control EVs, providing strong evidence for the selective uptake of these vesicles by myoFbs.

MiR-29 inhibits cardiac fibrosis through multiple signaling pathways, including the TGF-β, MAPK, and Wnt pathways. Despite its potential, no miR-29-based drugs are currently available for clinical use [18]. The primary challenge for miR-based therapy is the development of an efficient and stable cellular delivery system. Compared with other gene delivery vehicles, EVs are less immunogenic, noncytotoxic, and nonmutagenic. Consequently, EVs have been used therapeutically to deliver vaccines, chemotherapeutic drugs, miRNAs, and small interfering RNAs [11,30]. In this study, we developed Agomir-29b, a next-generation miR-29b mimic with enhanced stability and targeted delivery potential, and loaded it into αFAP-EL@CLD. Agomir-29b is a specially labeled and chemically modified double-stranded small RNA that mimics endogenous miRNA, exhibiting higher affinity for the cell membrane and greater enrichment than unmodified miRNA mimics, making it particularly suitable for in vivo interference experiments [31]. Our data demonstrated that αFAP-EL@CLD: Agomir-29b effectively targeted cultured myoFbs and fibrotic regions within the myocardium, significantly suppressing myoFb activation and alleviating ISO-induced cardiac fibrosis. Moreover, the required dosage of Agomir-29b was substantially lower when administered via αFAP-EL@CLD compared to free Agomir-29b administered intravenously.

We also investigated the TGF-β1 receptor inhibitor GW788388, known to block TGF-β1-induced Smad activation, thereby reducing cardiac fibrosis [22,23]. αFAP-EL@CLD loaded with GW788388 (αFAP-EL@CLD: GW788388) demonstrated substantial inhibition of myoFb activation and cardiac fibrosis progression, achieving therapeutic effects comparable to those of routine doses of GW788388. The heart-targeted accumulation of drug-loaded αFAP-EL@CLD resulted in high local drug concentrations, contributing to its pronounced therapeutic effects. Additionally, αFAP-EVs themselves contributed to the therapeutic advantages of αFAP-EL@CLD. EVs protected miR-29b from hydrolysis by RNase I, enhancing the stability of Agomir-29b in vivo. Furthermore, it has been reported that EV-encapsulated chemotherapeutic agents can impede drug clearance. Thus, αFAP-EL@CLD not only serves as drug delivery vehicles but also possesses unique features that significantly enhance the therapeutic efficacy of loaded drugs.

Although miR-29b and GW788388 have well-documented roles in treating cardiac fibrosis, with miR-29b targeting key pathways such as the TGF-β2, MMP2, and ERK/MAP kinase pathway and GW788388 inhibiting TGF-β type I receptor kinase activity to attenuate fibrosis, our primary focus in this study was not to further explore their established mechanisms. Instead, we aimed to demonstrate the efficacy of an EV-based drug delivery system, specifically targeting myoFbs, which represents the novel aspect of our work. These drugs were selected based on their proven efficacy in treating fibrosis, with the goal of investigating their delivery via a more efficient and stable EV-based system.

In this study, we focused on the development of an efficient and stable EV-based drug delivery system, specifically targeting myoFbs, rather than exploring the well-documented mechanisms of miR-29b and GW788388 in treating cardiac fibrosis. miR-29b is known to target key pathways such as the TGF-β2, MMP2, and ERK/MAP kinase pathways, while GW788388 inhibits TGF-β type I receptor kinase activity to attenuate fibrosis. These drugs were selected based on their proven efficacy in treating fibrosis, with our primary aim being to investigate their delivery through a novel EV-based system. Our data demonstrated that αFAP-EL@CLD vesicles enhanced drug accumulation in the fibrotic myocardium. When loaded with Agomir-29b or GW788388, these vesicles specifically delivered drugs to the heart, producing potent therapeutic effects against cardiac fibrosis. Importantly, the use of drug-loaded αFAP-EL@CLD is promising for clinical applications due to its excellent biological safety, high production yield, and simple production process. Consequently, αFAP-EL@CLD loaded with therapeutic agents represents a promising strategy for the treatment of fibrotic diseases.

## Data Availability

All data generated or analyzed during this study are included in this article.
