# Supplementary material for: Myofibroblast-Targeting Extracellular Vesicles: A Promising Platform for Cardiac Fibrosis Drug Delivery
Source: Biomater Res. 2025 Apr 11;29:0179. doi: 10.34133/bmr.0179 (PMC11986206; doi:10.34133/bmr.0179)
Supplement: Supplementary 1 — Figs. S1 to S5 [file bmr.0179.f1.docx]

**Supplementary Figure 1**

The sequences of αFAP chimeric protein, which consists of 307 amino acids with calculated molecular weight of 32kDa.

Color: Red, leader sequence; yellow, αFAP –scFv; green, linker domain; pink, transmembrane sequence; black, flag.

MPMGSLQPLATLYLLGMLVASCLGQVQLKESGGLVQPGGSLKLSCAASGFTFSSYGMSWVRQTADKRLELVATTNNNGGVTYYPDSVKGRFTISRDNAKNTLYLQMSSLQSEDTAMYYCARYGYYAMDYWGQGISVTVSSGGGSGGGSGGGSDVLMTQTPLWLPVSLGDQASISCRSSQSIVHSNGNTYLEWYLQKPGQSPKLLIYKVSNRFSGVPDRFSGSGSGTDFTVKISRVEAEDLGVYYCFGGSHVPYTFGGGTKLEIKGGGSGGGSGGGSSIISAVVGILLVVVLGVVFGILIDYKDDDDK

**Supplementary Figure 2**


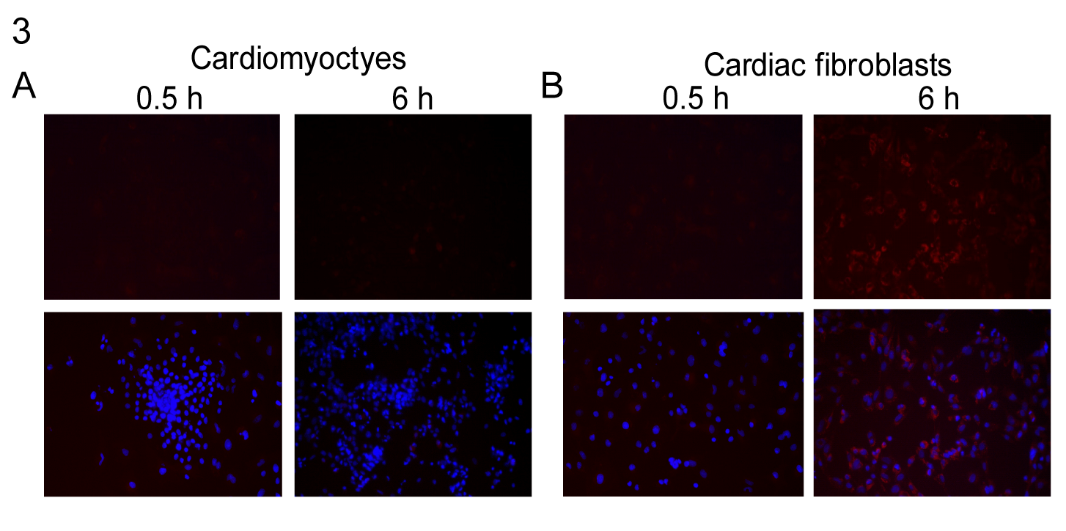


**Targeted delivery of αFAP-EVs into activated myofibroblasts in vitro.** Confocal fluorescence analysis of DiD uptake in NRCMs (A) and NRCFs (B) treated with 100 μg/mL DiD-labeled αFAP-EVs at the indicated time points (n=3).

**Supplemenary Figure 3**


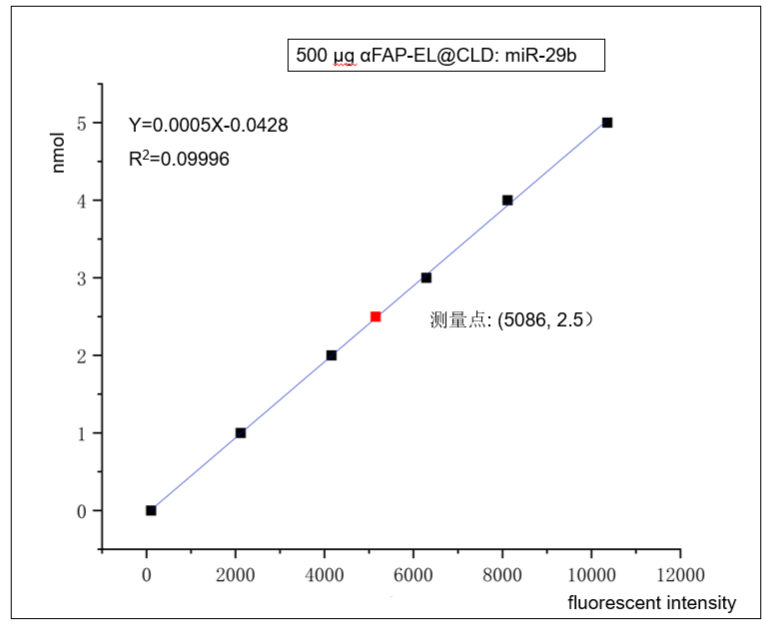


**The encapsulation efficiency of αFAP-EL@CLD for Agomir29b.** The standard curve for fluorescent intensity and miR29b mass.

**Supplementary Figure 4**


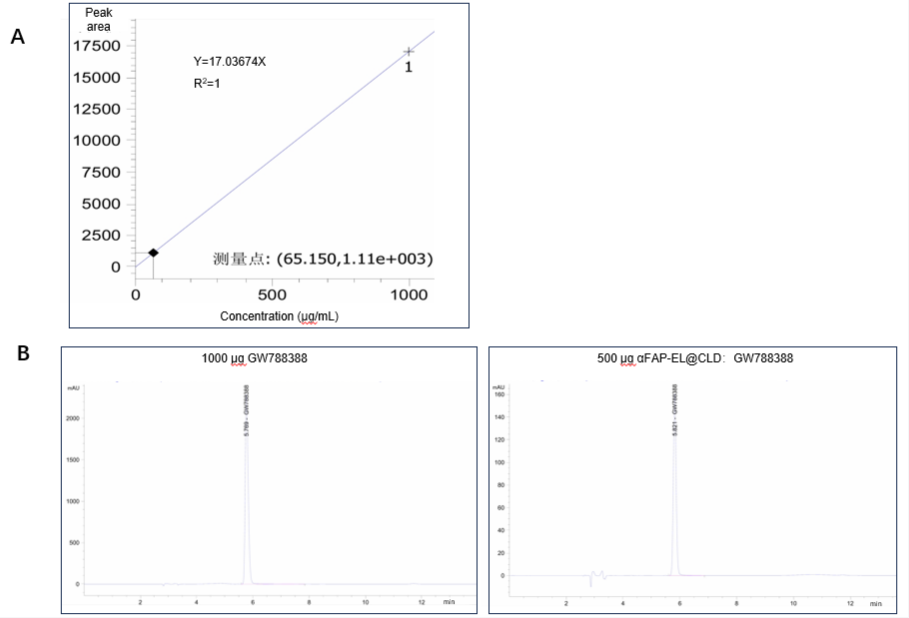


**The encapsulation efficiency of αFAP-EL@CLD for GW788388.** (A) Standard curve of the GW788388 concentration and the corresponding peak area obtained by HPLC analyses. (B) Representative results for 1000 μg GW7888388 standard and 500 μg αFAP-EL@CLD: GW7883388 analyzed by HPLC.

**Supplementary Figure 5**


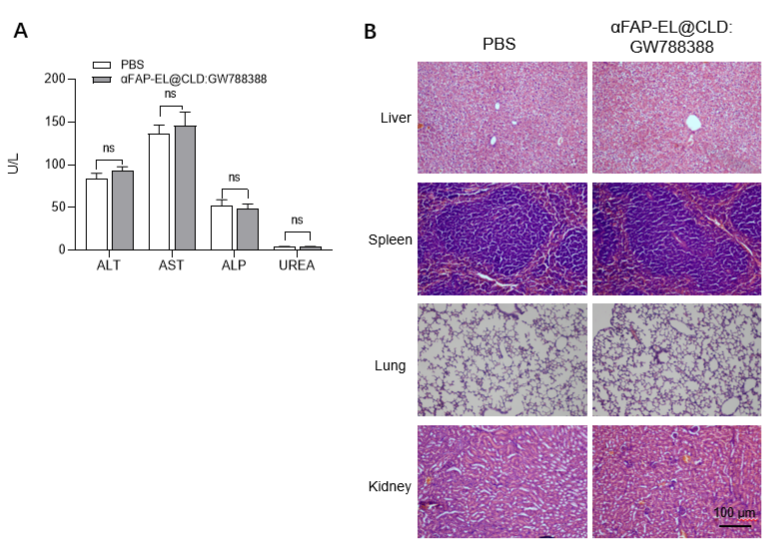


**The long-term biological safety effects of αFAP-EL@CLD: GW7883388.**

(A) Mice were intravenously injected with 100 μg αFAP-EL@CLD: GW788388 (≈ 2.3 × 10^10^particles) on days 3, 6 ,9 and 12 and were sacrificed on day 35. The levels of ALT, AST, ALP and UREA in sera were measured. (B) Histopathological damage in the liver, spleen, lungs and kidneys was detected by H&E staining.
